# A Report of Two Simultaneous Different Skull Vault Boney Pathologies: An Extremely Rare Clinical Scenario

**DOI:** 10.7759/cureus.40248

**Published:** 2023-06-11

**Authors:** Waleed F Dabbas, Mohammad Y Hiasat, Bilal Ibrahim, Razan Allababede, Tareq A Alkhaldi, Ayah Al warawrah, Mustafa Nadi

**Affiliations:** 1 Division of Neurosurgery, Department of Special Surgery, Faculty of Medicine, Al-Balqa Applied University, Al-Salt, JOR; 2 Department of Neurological Surgery, Neuron Clinics, Amman, JOR

**Keywords:** two pathologies, skull vault tumor, neurosurgery, skull hemangioma, osteoma

## Abstract

Primary calvarial boney tumors are generally rare in clinical practice. Multiple primary skull neoplasms are less frequent, typically associated with genetic disorders or familial syndromes. Sporadic cases of multiple skull tumors are exceptionally rare. We present a unique scenario of a 32-year-old female patient who had two right-sided skull vault lesions, one located over the right parietal area and the other in the right retro-auricular region. The lesions exhibited different behaviors over several years. The workup revealed that the two skull lesions were of two pathologies. The standard academic approach for clinical analysis attributes the symptoms often to one pathological process until proven otherwise. This case highlights the significance of expanding the differential diagnoses and incites clinicians to consider multiple pathologies in specific clinical settings.

## Introduction

Skull tumors are uncommon neoplasms, accounting for approximately 2% of musculoskeletal tumors [1]. Among benign primary skull tumors, osteoma is the most frequently encountered type. Skull bone hemangioma constitutes about 10% of benign primary skull tumors [2]. Instances of simultaneous growth of two distinct skull vault tumors are exceedingly rare, particularly in non-hereditary cases. In this particular case report, a patient underwent surgical treatment for two different histological skull lesions, and genetic analysis revealed no genetic disorders or familial syndrome. This case underscores the importance of a systematic clinical approach and widening the differentials in diagnosing and managing complex cases that involve multiple skull tumors. Achieving an accurate diagnosis and implementing appropriate treatment strategies play a crucial role in effectively managing these lesions.

## Case presentation

**Figure 1:**
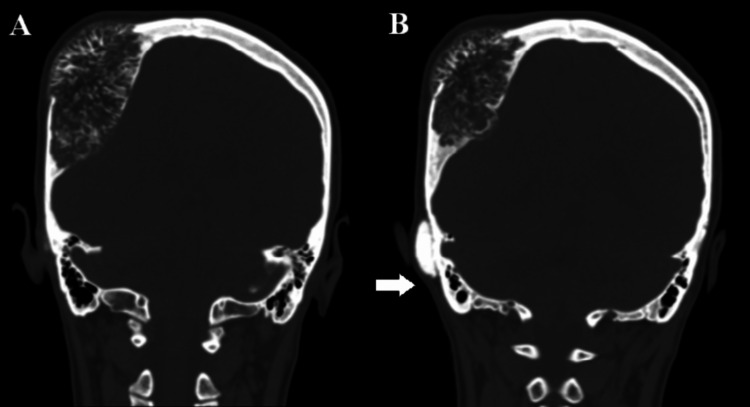
Brain CT scan bone window (A) Coronal view showing right parietal sunburst appearance of a bone tumor: a radiographic pattern that happens when the rate of lesion growth exceeds the periosteum capacity to lay down Sharpey’s fibers (collagen fibers that tie periosteum down to the bone). This results in splaying these fibers in radiating fashion. (B) Another coronal view showing the right parietal bone tumor and another expansile bone tumor arising from the outer cortex of the right mastoid part of the temporal bone (marked by a white arrow).

A 32-year-old female patient presented to our neurosurgery clinic with two right-sided scalp swellings over the right parietal and retro-auricular regions. She had no previous history of trauma, no significant past medical or surgical history, and no family history of bone or soft tissue tumors. The first mass was noticed in the right retro-auricular region when she was a teenager, causing no other symptoms. At that time, she underwent a brain computed tomography (CT) scan, and the mass was labeled as an osteoma arising from the mastoid part of the temporal bone. No further treatment was recommended. Four years later, she underwent a follow-up brain CT scan, and the previous mass did not show any significant changes. However, she was found to have another mass in the right parietal bone. Eight years later, when she became pregnant, the patient reported that the parietal mass was getting larger in size, but the retro-auricular mass remained the same. Five years later, the parietal lesion became larger and was associated with continuous right ear tinnitus, right-sided headache, and disfiguration of her skull shape. Her family history was negative for genetic diseases or bone tumors. After performing a brain CT scan, she was found to have a small right mastoid osteoma with an ipsilateral large parietal cavernous hemangioma (Figure 1, Panels A and B).

**Figure 2:**
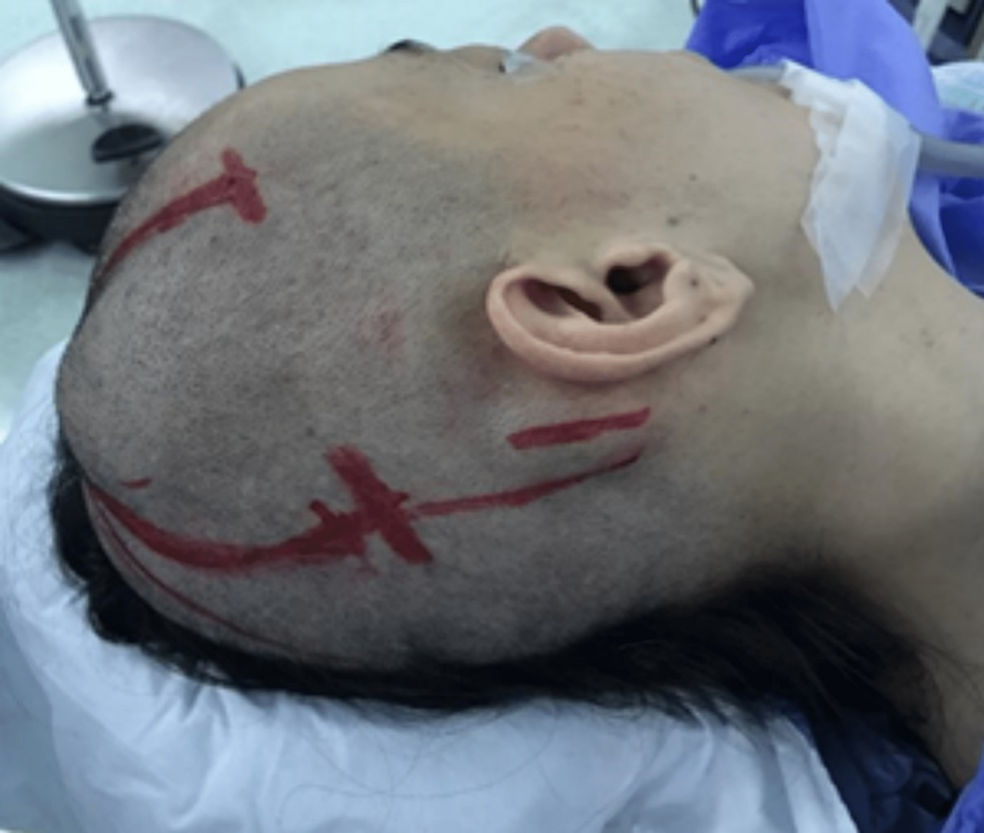
Patient's head positioning The patient was placed in a supine position with the head slightly elevated and rotated to the left side. Her right shoulder is elevated with a roll beneath it. Note the disfigurement caused by the right parietal mass. The skin incision was fashioned to suit the two lesions.

Surgical intervention was suggested and planned. Following the administration of general anesthesia and preoperative antibiotics, the patient was positioned supine with the head slightly elevated and rotated to the left side. A roll was placed beneath the right shoulder to ensure proper support and prevent pressure injuries, and all pressure points were adequately padded (Figure 2).

**Figure 3:**
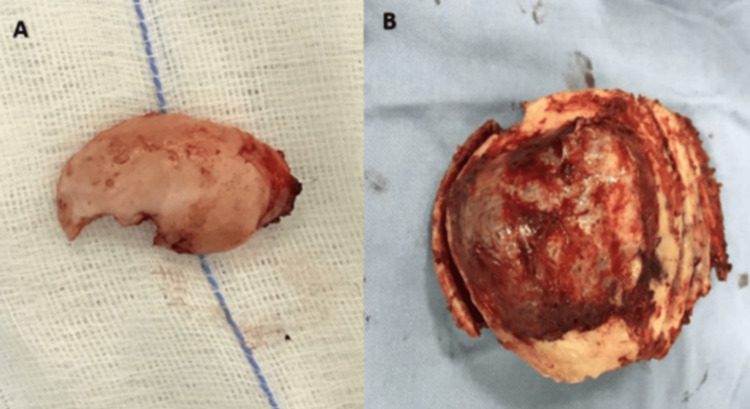
The bone neoplasms that were surgically excised (A) Retro-auricular osteoma measuring 3.5 cm x 2 cm x 1.5 cm excised completely en block from the outer plate of the mastoid bone. (B) Right parietal cavernous hemangioma measuring 10 cm x 8 cm x 4 cm, which looked completely excised. The histopathological examination confirmed the presence of slightly >1 cm safe margins around the hemangioma.

A question mark-shaped incision encompassing both lesions was marked, with the distal tip extending to the right mastoid region. The surgical area was then prepared and draped. The incision site was infiltrated with local anesthetic lidocaine 1% containing epinephrine (1:200,000). The scalp was meticulously opened in layers, and the pericranium was gently detached from the outer surface of the parietal mass and the surrounding normal bone, maintaining a safe margin. Subsequently, the mastoid mass was exposed and excised in one piece, including the outer table of the mastoid bone, using a chisel (Figure 3, Panel A). The tumor bed was carefully shaved using a high-speed drill. Two burr holes, located 2 cm away from the outer boundary of the parietal mass, were created on opposite poles. A craniotomy that included the lesion was performed by connecting the two burr holes with a high-speed drill. The mass was then separated and gently lifted from the underlying dura mater, which remained unaffected. A bone flap measuring 10 cm x 8 cm x 4 cm, inclusive of the mass, was excised as one piece (Figure 3, Panel B).

A synthetic flap made of methyl methacrylate was fashioned and used to reconstruct the skull defect. Titanium burr-hole cover plates, mini plates, and mini screws were employed to secure the new synthetic flap to the surrounding normal bone. The galea was reapproximated, and the scalp was closed in layers. The patient experienced an uneventful postoperative recovery.

**Figure 4:**
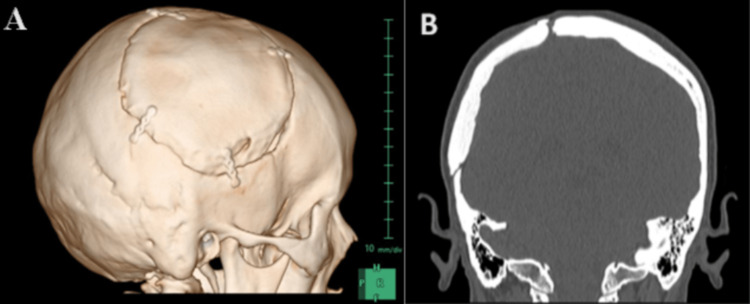
Follow-up head CT scan taken four years after tumor excision and cranioplasty (A) 3D reconstructed CT head. (B) Coronal view. Both images demonstrate the right parietal cranioplasty with no recurrence of either of the bone tumors.

The patient underwent regular follow-up visits in the initial year after the surgery and subsequently annually. Her follow-up was conducted over a four-year period, during which there were no indications of recurrence observed, both in terms of clinical examination and radiological assessments (Figure 4, Panels A and B).

**Figure 5:**
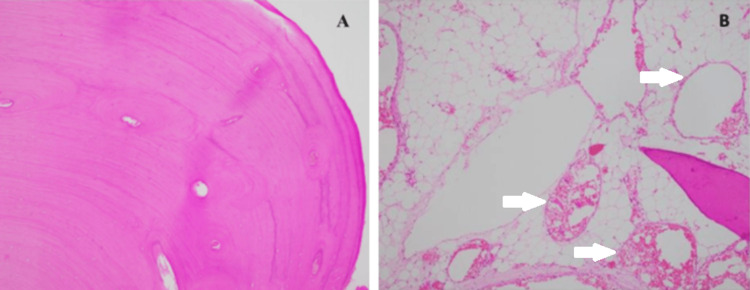
Hematoxylin and eosin stain for both boney lesions (H&E magnification power Xl00) (A) The picture illustrates a bony lesion composed of packed lamellar bone consistent with osteoma. (B) The slide shows a trabecular bone on the right-hand side's lower angle, which is surrounded by a fatty stroma containing many irregularly dilated large vascular spaces (white arrows) representing a cavernous type of hemangioma. The vascular spaces are lined by flat benign endothelial cells; some of the blood vessels in the lower part of the picture are filled with red blood cells.

Pathological gross examination of the right retro-mastoid lesion showed a bony piece measuring 3.5 cm x 2.5 cm x 1 cm. Histological examination of this lesion showed densely compact lamellar bone arranged in layers, with very minimal marrow spaces, consistent with the diagnosis of skull osteoma (Figure 5, Panel A). The gross examination of the right parietal mass revealed an oval piece of bone measuring 10 cm x 8 cm x 4 cm. Upon sectioning the specimen, a highly vascular lesion measuring 7 cm x 5 cm x 3 cm was observed, indicating complete excision of the lesion. Microscopic examination of the sample shows a trabecular bone surrounded by fatty stroma and dilated vascular spaces. The vascular element is characterized by flat endothelial cells lining and filled with red blood cells. These histologic features represent a cavernous type of skull hemangioma (Figure 5, Panel B).

Additionally, the Ki67 proliferative index was determined to be 8%. The patient underwent genetic testing, which included a panel of tens of genes from different syndromes, including Gardner's syndrome for APC (Adenomatous polyposis coli) gene mutation (a tumor suppressor gene), and the results were negative. There was no history of familial syndromes or genetic disorders in the family.

## Discussion

Skull bone tumors (osteomas and cavernous hemangiomas) are rare human neoplasms, accounting for less than 2% of all musculoskeletal malignancies [1,2]. The skull vault comprises the inner and outer cortical tables as well as the marrow space between them [3,4]. The most common primary skull tumor is benign osteoma [1]. Skull vault osteomas are osteogenic neoplasms of the cancellous bone, typically presenting as solitary lesions in the head and neck [2]. They consist of a combination of compact and trabecular bone and usually grow near the outer table of the skull bone, either as sessile or pedunculated tumors [5]. However, in rare cases, they may arise from the inner table or within the diploe, known as intradiploic osteoma [6].

Osseous hemangioma is a slowly growing benign vascular bone tumor, accounting for less than 1% of primary bone lesions and 10% of benign calvarial tumors [7-9]. The frontal and parietal bones are the most common sites for this benign lesion [8,10]. Osseous hemangiomas are predominantly seen in individuals over 30 years old and are slightly more common in females [7]. Most osseous hemangiomas exhibit a cavernous type, characterized by dilated blood vessels separated by fibrous septa, while a minority of cases display a capillary feature without dividing fibrous septa [8,11]. Although solitary lesions are more common, several cases of multiple osseous hemangiomas have been reported [8-10].

The synchronous occurrence of different benign skull tumors is extremely rare, particularly in sporadic cases. However, multiple skull tumors are associated with certain syndromes. For instance, Gardner's syndrome, an autosomal dominant inherited disease and a form of familial adenomatous polyposis, can present with multiple skull osteomas [12]. Ollier's disease and Maffucci's syndrome, both characterized by the co-occurrence of multiple chondromas within the metaphyseal plate, may also involve the skull [13]. Neurofibromatosis type 1 is associated with giant cell granulomas that can affect the skull and facial bones [14].

In the present case, surgical resection was performed to remove two skull lesions with different pathological characteristics. The gross and microscopic examinations provided valuable insights into the nature and features of these lesions. The parietal mass was confirmed as a cavernous hemangioma, exhibiting a highly vascular nature. Cavernous hemangioma increased in size and became more symptomatic during pregnancy, which could be attributed to several factors. First, the increase in the intravascular volume and associated hemodynamic changes during pregnancy could result in vascular channel expansion within the hemangioma. Second, the hormonal fluctuation namely high progesterone level can augment vascular volume and high estrogen can upregulate endothelial proliferation [15]. The retro-mastoid lesion was identified as an osteoma based on microscopic analysis. Furthermore, the Ki67 proliferative index of 8% indicated a low level of cell proliferation within the cavernous hemangioma. This can provide information about its growth potential, and subsequently, less aggressive follow-up might be required at the Ki67 index < 10%. The patient does not have any other osseous lesions in her body. She has no signs of Ollier’s disease or Maffucci syndrome. Genetic analysis revealed no familial or genetic syndromes associated with the patient.

Regular follow-up examinations showed no evidence of recurrence for the excised lesions. The clinical behavior of sporadic cases of surgically managed multiple skull lesions, whether benign or malignant, remains largely unknown. However, in familial cases, many skull tumors have shown a more malignant course [16-18]. Furthermore, our extensive search on PubMed and Google Scholar did not yield any similar cases for comparison or reference. This highlights the need for further research to understand the clinical behavior of sporadic cases involving multiple skull tumors, specifically in determining whether they exhibit benign or malignant characteristics. Additionally, it is crucial to investigate how the behavior of these tumors might differ if they were to develop as solitary tumors instead of appearing synchronously. Additional studies and investigations are necessary to address these important questions and enhance our understanding of these rare and complex conditions.

## Conclusions

We presented a case with two skull masses, where both radiological and histological findings confirmed the simultaneous presence of a bony cavernous hemangioma and calvarial osteoma. While conventional academic case analysis typically attributes symptoms to a single pathology, clinicians should broaden their differentials in complex clinical scenarios. This case report underscores the importance of scrutinizing intricate cases with multiple skull lesions. Accurate diagnosis and appropriate treatment are essential in effectively managing these cases.
